# Human Rhinovirus Infections in Rural Thailand: Epidemiological Evidence for Rhinovirus as Both Pathogen and Bystander

**DOI:** 10.1371/journal.pone.0017780

**Published:** 2011-03-29

**Authors:** Alicia M. Fry, Xiaoyan Lu, Sonja J. Olsen, Malinee Chittaganpitch, Pongpun Sawatwong, Somrak Chantra, Henry C. Baggett, Dean Erdman

**Affiliations:** 1 Influenza Division, Centers for Disease Control and Prevention, Atlanta, Georgia, United States of America; 2 Division of Viral Diseases, Centers for Disease Control and Prevention, Atlanta, Georgia, United States of America; 3 Division of Emerging Infections and Surveillance Services, Centers for Disease Control and Prevention, Atlanta, Georgia, United States of America; 4 National Institute of Health, Thailand Ministry of Public Health, Nonthaburi, Thailand; 5 International Emerging Infections Program, Thai MOPH-U.S.CDC Collaboration, Nonthaburi, Thailand; University of Hong Kong, Hong Kong

## Abstract

**Background:**

We describe human rhinovirus (HRV) detections in SaKaeo province, Thailand.

**Methods:**

From September 1, 2003–August 31, 2005, we tested hospitalized patients with acute lower respiratory illness and outpatient controls without fever or respiratory symptoms for HRVs with polymerase chain reaction and molecularly-typed select HRVs. We compared HRV detection among hospitalized patients and controls and estimated enrollment adjusted incidence.

**Results:**

HRVs were detected in 315 (16%) of 1919 hospitalized patients and 27 (9.6%) of 280 controls. Children had the highest frequency of HRV detections (hospitalized: <1 year: 29%, 1–4 year: 29%, ≥65 years: 9%; controls: <1 year: 24%, 1–4 year: 14%, ≥65 years: 2.8%). Enrollment adjusted hospitalized HRV detection rates were highest among persons aged <1 year (1038/100,000 persons/year), 1–4 years (457), and ≥65 years (71). All three HRV species were identified, HRV-A was the most common species in most age groups including children aged <1 year (61%) and all adult age groups. HRV-C was the most common species in the 1–4 year (51%) and 5–19 year age groups (54%). Compared to controls, hospitalized adults (≥19 years) and children were more likely to have HRV detections (odds ratio [OR]: 4.8, 95% confidence interval [CI]: 1.5, 15.8; OR: 2.0, CI: 1.2, 3.3, respectively) and hospitalized children were more likely to have HRV-A (OR 1.7, CI: 0.8, 3.5) or HVR-C (OR 2.7, CI: 1.2, 5.9) detection.

**Conclusions:**

HRV rates were high among hospitalized children and the elderly but asymptomatic children also had substantial HRV detection. HRV (all species), and HRV-A and HRV-C detections were epidemiologically-associated with hospitalized illness. Treatment or prevention modalities effective against HRV could reduce hospitalizations due to HRV in Thailand.

## Introduction

Human rhinoviruses (HRVs) are members of the family *Picornaviridae*, genus *Enterovirus*, and comprise 3 species, HRV-A, HRV-B and the recently recognized HRV-C [1], [2], [3]. HRVs are a well established cause of the common cold, an upper respiratory illness characterized by rhinorrhea, nasal congestion, cough, sore throat and sneezing; more than half of upper respiratory illnesses are caused by HRVs [4]. Recently, several studies have suggested that HRVs may be associated with more severe illness, including hospitalized lower respiratory disease and asthma exacerbations [5], [6] [7], [8], [9], [10], [11], [12], [13], [14], [15]. Also, some studies suggest that infection with HRV-C may result in more severe illness compared to illness with other species [1], [2], [16], [17].

HRVs have also been identified among asymptomatic persons (detection in the nasopharynx in the absence of respiratory symptoms) as an innocent “bystander virus” [18]. The reported prevalence of HRV detection among asymptomatic persons ranges from 12–22% among children and 9% among adults with immunocompromising conditions [19], [20], [21], [22]. Consequently, the clinical relevance of detection of HRV among hospitalized patients can be difficult to interpret and better studies are needed to ascertain the role of HRVs as pathogens to spur efforts to develop treatment and prevention modalities. To that end, we took advantage of a study in rural Thailand that concurrently enrolled hospitalized patients with acute lower respiratory illness, a sample of outpatients with influenza-like illness, and control outpatients without fever or respiratory symptoms from the same hospitals to better define the etiologic role of HRV infection and associated disease burden in this community.

## Methods

The surveillance system and laboratory procedures have been published previously [23]. Briefly, residents of Sa Kaeo province, Thailand who were identified in active population-based surveillance for hospitalized acute respiratory illness were approached for enrollment into a pneumonia etiology study during September 1, 2003 through August 31, 2005. Enrollment occurred in all 8 hospitals in Sa Kaeo province. A patient was eligible for enrollment if he/she had at least one sign of acute infection (e.g. fever, abnormal white blood cell count) plus signs or symptoms of lower respiratory tract disease (e.g. cough, abnormal breath sounds) and the physician ordered a chest radiograph within 48 hours of admission. Each enrolled patient had a nasopharyngeal swab, acute and convalescent serum, and demographic information collected. We considered repeat hospitalizations that occurred ≥14 days after the previous discharge date as individual hospitalizations. Chest radiographs were read by an expert panel as described previously [24].

In addition, a convenience sample of outpatients with influenza-like illness (fever plus cough or sore throat) was recruited from the outpatient departments from the same hospitals in Sa Kaeo Province during the study period [25]; 8 outpatient departments participated during year one and 5 departments participated during the following year. Each outpatient participant had a nasopharyngeal swab and demographic information collected. During the second study year, September 1, 2004–August 31, 2005, we collected nasopharyngeal swabs and demographic information from control outpatients (i.e., patients who did not have a fever, and did not report a fever, cough, sore throat or diarrhea within the previous 3 days) from the outpatient clinics as described above. We sought to enroll the same number of controls from five age groups (≤2 yrs, 3–5 yrs, 6–15 yrs, 16–54 yrs, and ≥55 yrs) during each month of the year, (median number/month for all controls = 25, range 10–30).

The processing and testing of nasopharyngeal swabs were described elsewhere and included individual PCR assays for each pathogen and viral isolation for some viruses [23]. All specimens were tested by a laboratory blinded to patient status, e.g., hospitalized, outpatient, or control. Nasopharyngeal specimens were tested for respiratory syncytial virus (RSV), human metapneumovirus (HMPV), human parainfluenza viruses (HPIVs) 1, 2, and 3, influenza viruses A and B, adenovirus, *Legionella* species, *Chlamydia pneumoniae*, and *Mycoplasma pneumonia*, and rhinovirus/enterovirus (Table S1). HRV was detected from respiratory specimens by first testing with RT-PCR primers to detect rhinovirus/enterovirus as described in Roghmann, et al [26]. To discriminate between HRVs and human enteroviruses, PCR positive samples were retrospectively subjected to a second round of semi-nested PCR using HRV-specific primers as described in Miller, et al. [11]. Viral isolation was performed for RSV, HPIV, influenza viruses and adenovirus. Paired sera was tested for RSV, HMPV, HPIV 1,2 and 3, adenovirus, influenza, *Legionella* sp., *Chlamydia penumoniae*, and *Mycoplasma pneumoniae* as described [23], [27]. For HRV, a positive RT-PCR result was considered a positive result. For other pathogens, a positive culture, RT-PCR, or four-fold rise in serology was considered a positive test result. Serologic testing and PCR assays for *Legionella* sp., *Chlamydia penumoniae*, and *Mycoplasma pneumoniae* were only performed on patients enrolled during the first study year, September 2003–August 31, 2004 and interpreted as previously described [27]. In addition, diagnostic testing included urine pneumococcal surface antigen assay among pneumonia patients aged ≥18 years. HRV positive samples from hospitalized and control patients during September 1, 2004 through August 31, 2005 were further identified to species by RT-PCR and sequencing of partial region of the VP1 (D. Erdman, unpublished, method available on request). Specimens that were VP1 negative were further tested using primers that amplified partial VP4/VP2 or 5′NCR regions [28].

We calculated crude and age-specific (<1, 1–4, 5–19, 20–49, 50–64, ≥65 years) incidence for hospitalized HRV infections using population estimates from Thailand's National Economic and Social Development Board for the period January 1, 2003 through December 31, 2005 [29]. Because not all patients eligible for our study were enrolled, we estimated adjusted incidence to account for eligible patients who did not enroll. For the adjusted incidence we assumed the proportion of HRV detections in the enrolled patients was the same as the proportion of infections among all eligible patients.

To determine whether HRV detections were epidemiologically associated with hospitalized respiratory illness, we compared HRV detections among hospitalized enrolled patients to detections among control patients without fever or respiratory symptoms. Because controls were collected from the second study year, September 1, 2004–August 31, 2005, this comparison was limited to hospitalized patients from the same year. Two-tailed *P* values of <0.05 were considered to be statistically significant.

All participants were informed of the study objectives and written consent was obtained. This study protocol was reviewed and approved by the Centers for Disease Control and Prevention (CDC) Internal Review Board and the Thailand Ministry of Public Health Internal Review Board.

## Results

From September 1, 2003 to August 31, 2005, 7449 patients with signs and symptoms of acute lower respiratory tract infection were admitted to hospital in Sa Kaeo province and 4347 (58%) of these patients had a chest radiograph ordered within 48 hours. We enrolled 1941 (45%) of the eligible hospitalized patients (e.g. with acute lower respiratory illness and a chest radiograph). Among all eligible patients, those who were and were not enrolled had similar presenting signs and symptoms (data not shown), intubation (4.2% vs. 5%), and fatalities (4% vs. 5.2%), respectively. However, persons who were enrolled were older compared to those not enrolled (median age 40 years (interquartile range (IQR): 3–67 years) versus 20 years (IQR: 2–63 years), respectively). In addition, we enrolled a sample of 1569 outpatients with influenza-like illness. During the second year of the study, we enrolled 280 control patients.

We detected HRVs in 16% of hospitalized enrolled patients (overall and with radiographically-confirmed pneumonia), 19% of outpatients with influenza-like illness, and 9.6% of control patients (Table 1). The frequency of HRV detection was highest among enrolled patients ≤19 years of age, especially young children aged <5 years. However, a substantial proportion of hospitalized adult patients in all age groups had HRVs detected. Among adults >20 years of age with radiographically-confirmed pneumonia, 7.9% had HRV detection. Among all patients with radiographically-confirmed pneumonia, 16% had HRV detection. The proportion of HRV positive control patients was highest among the youngest children; 4 (14%) of 28 infants aged 0–6 months and 4 (67%) of 6 infants aged 6–11 months had HRV detections. Among all patients, HRV detections were statistically more common among hospitalized enrolled patients compared to controls. However, there were differences in the association between hospitalization and HRV detection when stratified by age group, evidence for effect modification by age.

**Table 1:** Comparison of HRV detections among hospitalized patient with lower respiratory tract infections, outpatients with influenza-like illness and controls£ without respiratory symptoms and fever in the previous 3 days, Sa Kaeo Province, Thailand, September 1, 2003–August 31, 2005.

Age groups	Hospitalized EnrolledHRV+/Total enrolled (%)	Hospitalized CXRPneumoniaHRV+/Total CXR pneumonia (%)	Outpatients with ILIHRV+/Total enrolled (%)	Controls*HRV+/Total enrolled (%)	OR£ (95% CI)
0–11 mo	47/164 (29)	21/103 (20)	27/95 (28)	8/34 (24)	1.4 (0.6, 3.4)
1–4 yrs	105/366(29)	69/249 (28)	145/594 (24)	7/51 (14)	2.3 (1.0, 5.2)§
5–19 yrs	37/182 (20)	20/80 (25)	103/706 (15)	9/69 (13)	1.8 (0.8, 4.0)
20–49 yrs	47/406 (11)	25/237 (11)	13/112 (12)	2/54 (3.7)	3.3 (0.8, 14.5)
50–64 yrs	32/273 (12)	20/172 (12)	4/41 (9.8)	0/36	§
≥65 yrs	47/545 (9)	25/317 (8.0)	2/21 (9.5)	1/36 (2.8)	2.9 (0.4, 22.0)
Total	315/1936 (16)	180/1158 (16)	294/1569 (19)	27/280 (9.6)	1.9 (1.2, 2.9)§

HRV = human rhinovirus, CXR = chest radiograph-confirmed, ILI = influenza-like illness, OR = crude odds ratio.

*Control patients were only enrolled during year two, Sept 1, 2004–Aug 31, 2005.

£ Hospitalized enrolled patients from year two, Sept 1, 2004–Aug 31, 2005, were compared to controls. The number of hospitalized patients with HRV detection during year two were 1–11 months: 31/103 (30%), 1–4 years: 73/276 (26%), 5–19 years: 26/125 (21%), 20–49 years (24/212 (11%), 50–64 years: 21/156 (13%), ≥65 years: 23/302 (7.6%). The results were similar with age groups stratified by ≤2 yrs (OR = 1.8, 95% CI 0.89, 3.8, p = .09), 3–5 yrs (OR = 1.2, 95% CI 0.42, 3.2, p = .75), 6–15 yrs (OR = 2.0, 95% CI 0.84, 4.9, p = .11), 16–54 yrs (OR = 4.0, 95% CI 0.94, 17.3, p = .04), and ≥55 yrs (OR = 6.7, 95% CI 0.90, 49.7, p = .03).

§ X^2^P≤.05.

The adjusted incidence rates of HRV detections among hospitalized patients with lower respiratory tract illness were highest among children <1 year, children 1–4 years, and adults ≥65 years of age (Table 2). Rates were high even after excluding patient with co-detections with other respiratory pathogens. Young children and elderly adults also had high rates of radiographically-confirmed pneumonia. Rates of hospitalized enrolled adults from Sa Kaeo province aged >50 years with HRV detections and lower respiratory tract illness and radiographically-confirmed pneumonia have been published previously (enrolled patients with [89/100,000] and without co-detection of another pathogen [78/100,000]; radiographically-confirmed pneumonia with [51/100,000] and without co-detection of another pathogen [39/100,000]) [23].

**Table 2:** Crude and enrollment adjusted age group specific incidence* for hospitalized HRV detections, Sa Kaeo Province, Sept 1, 2003–Aug 31, 2005.

	Hospitalized Enrolled			Hospitalized CXR Pneumonia		
Age groups	Crude	Adjusted for enrollment	Adjusted, excluding co-detections**	Crude	Adjusted for enrollment	Adjusted, excluding co-detections**
0–11 mo	323	1038	574	145	464	265
1–4 yrs	173	457	277	115	303	189
5–19 yrs	13	29	21	6.9	16	12
20–49 yrs	9.5	21	18	5.2	12	10
50–64 yrs	25	53	36	16	33	21
>65 yrs	75	159	139	40	85	74
Total	30	71	49	17	41	29

HRV = human rhinovirus, CXR = chest radiograph-confirmed,

*Rate/100,000 persons/year.

**Co-detections included RSV, HMPV, HPIV 1–3, influenza type A or B, and adenovirus for all patients, and *S. pneumoniae* for adults aged ≥18 years. During the first study year, co-detections with *Mycoplasma*, *Chlamydia*, and *Legionella* were excluded.

HRV co-detections with other respiratory pathogens were common among all age groups, but most striking among young children (Table 3). Among hospitalized enrolled patients with HRV detection: 30 (9.5%) were co-infected with RSV, 19 (3.5%) with HPIV, 16 (6.0%) with influenza A or B, 5 (1.6%) with HMPV and 20 (6.3%) with adenoviruses. Co-detection of pathogens for each age-group is detailed elsewhere^23^. Among 306 hospitalized HRV positive adults aged ≥18 years 15 (4.9%) had co-detection of *S. pneumonia*. During the first study year, eight (6.8%) of 117 patients with HRV had co-detection of an atypical bacterial pathogen [27], one adult with *L. longbeacheae*, two patients with *M. pneumoniae*, and five patients with co-detection of *Chlamydia pneumoniae*. Among outpatients with influenza-like illness and HRV detection: 7 (2.3%) were co-infected with RSV, 14 (4.6%) with HPIV 1, 2, or 3, 37 (12%) with influenza A or B, 14 (4.6%) with HMPV, and 16 (5.3%) with adenoviruses. Among controls, one had co-detection of HPIV-2 and one with adenovirus.

**Table 3:** Patients with HRV detection and co-detection of other respiratory pathogens*, Sa Kaeo Province, Thailand, September 1, 2003–August 31, 2005.

Age groups	Hospitalized Enrolled	Hospitalized CXR pneumoniaCo-detections/HRV+ (%)	Outpatients with ILICo-detections/HRV+ (%)	Controls£Co-detections/HRV+ (%)
1–11 mo	21/47 (45)	8/21 (43)	2/27 (11)	1/8 (13)
1–4 yrs	42/105 (40)	26/68 (38)	51/145 (35)	1/7 (14)
5–19 yrs	10/37 (27)	5/18 (28)	32/103 (31)	0/9
20–49 yrs	9/47 (19)	3/25 (12)	0/13	0/2
50–64 yrs	10/32 (31)	7/20 (35)	0/4	0
≥65 yrs	6/47 (13)	3/25 (12)	0/2	0/1
Total£	98/315 (31)	53/177 (30)	86/294 (29)	2/27 (7.4)

HRV = human rhinovirus, CXR = chest radiograph-confirmed, ILI = influenza-like illness.

*Co-detections included RSV, HMPV, HPIV 1–3, influenza type A or B, and adenovirus for all patients, and *S. pneumoniae* for hospitalized adults aged ≥18 years. During the first study year, co-detections with *Mycoplasma*, *Chlamydia*, and *Legionella* were included for hospitalized patients.

£ Control patients were only enrolled during the second study year, Sept 1, 2004–Aug 31, 2005.

All three HRV species were identified from the 225 HRV-positive specimens from the control and hospitalized patients enrolled during the second year of the study (Table 4); no statistically significant differences in the proportion of individual species between controls and hospitalized patients were found. HRV-A was the most common species in most age groups including children aged <1 year and all adult age groups. HRV-C was the most common species in the 1–4 year and 5–19 year age groups. Among hospitalized patients, co-detection of other respiratory pathogens did not vary by HRV species (HRV without co-detection: HRV-A: 56 [70%], HRV-B: 20 [77%], HRV-C: 48 [67%]) or by the presence of radiographically-confirmed pneumonia (HRV-A: 37 [46%], HRV-B: 14 [54%], HRV-C: 38 [52%]).

**Table 4:** HRV species identified among hospitalized enrolled patients and controls£, Sa Kaeo province, Thailand, September 1, 2004–August 31, 2005 (n = 229).

Patients with HRV	Species			
	A	B	C	Other**
Controls (n = 27), No. (%)	11(41)	5 (19)	9 (33)	2 (7.4)
Hospitalized (n = 198)	80 (40)	26 (13)	73 (37)	19 (10)
Hospitalized, by age group				
1–11 mo (n = 31)	19 (61)	1 (3.2)	7 (23)	4 (13)
1–4 yrs (n = 73)	25 (34)	3 (4.2)	37 (51)	8 (11)
5–19 yrs (n = 26)	5 (19)	3 (12)	14 (54)	4 (15)
20–49 yrs (n = 24)	12 (50)	6 (26)	5 (22)	1(4.2)
50–64 yrs (n = 21)	9 (43)	4 (19)	7 (33)	1 (4.8)
≥65 yrs (n = 23)	10 (43)	9 (39)	3 (13)	1 (4.4)

HRV = human rhinovirus.

£ Outpatients without respiratory symptoms and fever in the previous 3 days.

*X^2^ P = .001 comparing species across age groups.

**Species not identified.

We compared HRV detection among hospitalized patients and controls stratified into two groups, adult and children, due to possible effect modification by age (Table 5). After adjusting for month of detection, HRV detections were statistically more common among hospitalized enrolled adults and children compared to controls, the association was 2-fold elevated among adults. The association with hospitalization persisted when patients with co-detection of other respiratory pathogens were excluded. Detection of HRV- A and HRV-C, when other respiratory pathogen co-detections were excluded, were statistically associated with hospitalization among children. The risk of HRV- A and HRV-C detections among hospitalized adults compared to controls was elevated but not statistically significant. The associations were unchanged when we did not adjust for month of detection.

**Table 5:** Comparison of HRV detections among hospitalized enrolled patients and controls, Sa Kaeo Province, Thailand, September 1, 2004–August 31, 2005.

	HospitalizedNo./No. (%)	ControlsNo./No. (%)	Adjusted* OR(95% CI)
**Adults ≥19 years**			
All HRV detections	68/673 (10)	3/128 (2.3)	4.8 (1.5, 15.8)£
Excluding co-detections**	54/577 (9.4)	3/128 (2.3)	4.4 (1.3, 14.4)£
HRV-A	31/673 (4.6)	1/128 (0.8)	6.2 (0.8, 46.2)
Excluding co-detections**	25/577 (4.3)	1/128 (0.8)	5.6 (0.7, 42.4)
HRV-B	19/673 (2.8)	1/128 (0.8)	3.7 (0.5, 28.4)
Excluding co-detections**	14/577 (2.4)	1/128 (0.8)	3.0 (0.4, 23.6)
HRV-C	15/673 (2.2)	1/128 (0.8)	3.2 (0.4, 24.5)
Excluding co-detections**	12/577 (2.1)	1/128 (0.8)	3.0 (0.4, 23.6)
**Children <19 years**			
All HRV detections	130/501 (26)	24/152 (16)	2.0 (1.2, 3.3)£
Excluding co-detections**	78/322 (24)	22/150 (15)	2.1 (1.2, 3.6)£
HRV-A	49/501 (10)	10/152 (6.6)	1.7 (0.8, 3.5)
Excluding co-detections**	31/322 (10)	8/150 (5.3)	2.3 (1.0, 5.3)£
HRV-B	7/501 (1.4)	4/152 (2.6)	0.5 (0.1, 1.8)
Excluding co-detections**	6/322 (1.9)	4/140 (2.7)	0.7 (0.2, 2.7)
HRV-C	58/501 (12)	8/152 (5.3)	2.7 (1.2, 5.9)£
Excluding co-detections**	37/322 (11)	8/150 (5.3)	2.4 (1.0, 5.5)£

HRV = human rhinovirus, OR = odds ratio, CI = confidence interval.

*Adjusted for month of detection.

£ P≤.05.

**Co-detections included RSV, HMPV, HPIV 1–3, influenza type A or B, and adenovirus for all patients, and *S. pneumoniae* for hospitalized adults aged ≥18 years. During the first study year, co-detections with *Mycoplasma*, *Chlamydia*, and *Legionella* were included for hospitalized patients.

We looked for clinical evidence that co-detection of HRV with RSV might be similar to clinical illness associated with RSV and be different than illness associated with HRV only detections. We found no difference in clinical symptoms at the time of hospital admission among children aged <5 years with either RSV-HRV co-detection, RSV only, or HRV only detection, including frequency of fever, tachypnea, abnormal breath sounds and cough, and no differences in the proportion of children receiving mechanical ventilation or supplemental oxygen use (data not shown). However, children with HRV detection had fewer radiographically-confirmed pneumonias compared to patients with RSV detections (RSV: 100/135 [74%], HRV: 72/124 [58%], p<.05). There were no differences between RSV-HRV co-detections (18/28 [64%] and RSV or HRV alone. Five patients with HRV detected died while hospitalized during the study period; three were ≥65 years of age and none had co-detection of another respiratory pathogen.

We found no differences in clinical characteristics among hospitalized enrolled patients with HRV-A, -B or -C detections, including cough (A: 68/79 [85%], B: 21/26 [81%], C: 67/70 [96%]), temperature ≥38.2°C at admission (A: 47/79 [59%], B: 17/26 [65%], C: 36/71 [51%]), age-specific tachypnea (A: 15/78 [19%],B: 4/24 [17%],C: 14/68 [21%]), wheezing (A: 22/79 [28%], B: 9/26 [35%], C; 23/68 [34%]), elevated white blood cell count (>11,000) (A: 50/78 [64%], B: 15/26 [58%], C; 48/67 [72%]), ventilator use (A:3/78 [3.8%], B: 0/24,C; 2/72 [2.7%]), or in hospital deaths (A: 3/78 [3.7%], B: 1/26 [3.9%], C: 0/73).

**Figure 1:**
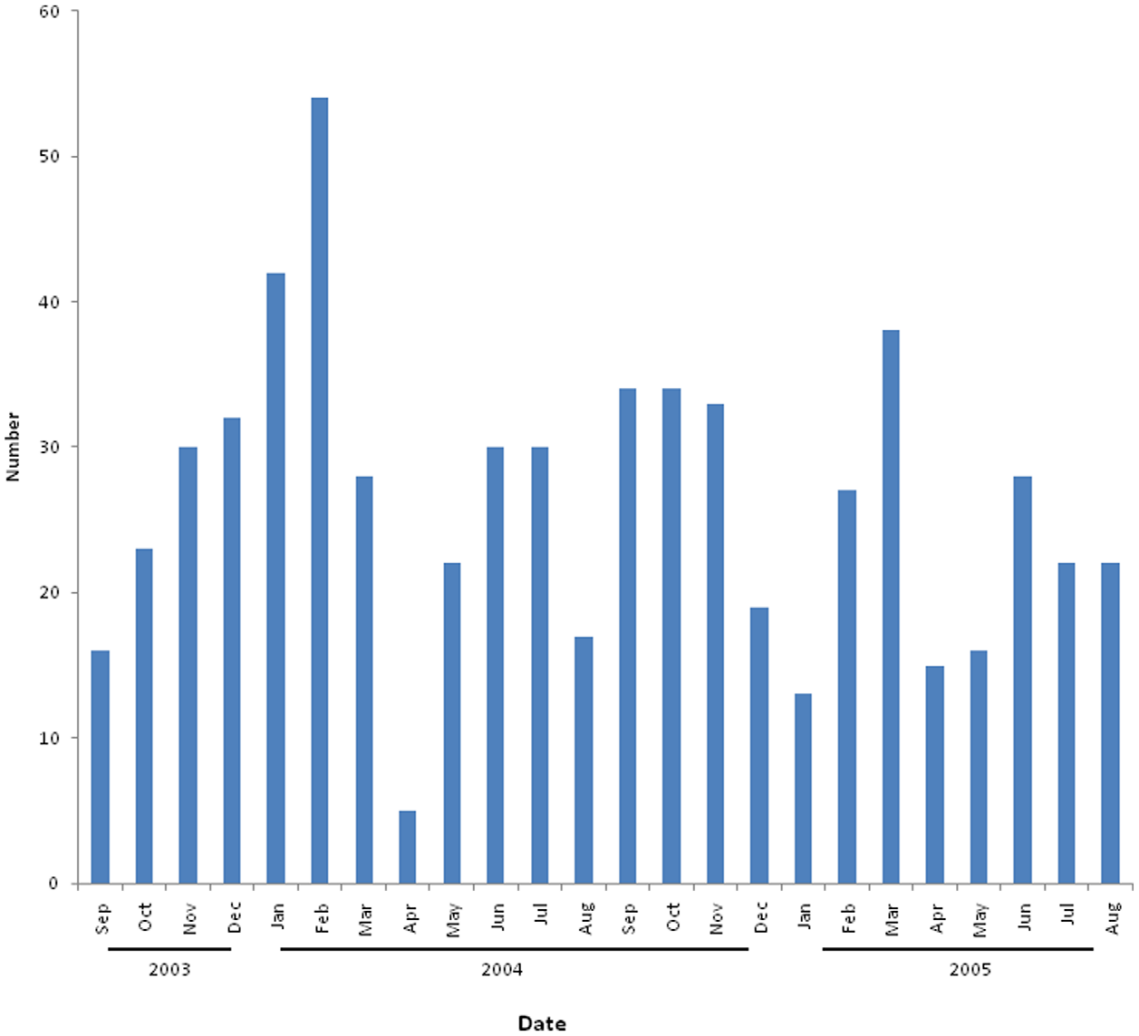
Seasonality of HRV detections among enrolled hospitalized patients and outpatients with influenza-like illness, Sa Kaeo Province, Sept 1, 2003–Aug 31, 2005.

HRVs were detected during all months of the year among all enrolled ill patients, including outpatients with influenza-like illness and hospitalized enrolled patients with lower respiratory tract illness, with some decrease in detections during the hot and dry months of April and May (Figure 1). HRV-A, -B and -C appeared to circulate concurrently with no clear temporal predominance of one species (data not shown).

## Discussion

We report a high proportion of HRV detections among hospitalized patients of all age groups in rural Thailand and evidence that HRV detections were epidemiologically associated with hospitalized lower respiratory tract disease among both children and adults. In addition, both HRV-A and HRV-C were common among hospitalized patients. Children aged <1 year and 1–4 years and adults ≥65 years of age had the highest rates of hospitalized HRV- associated acute lower respiratory illness and pneumonia.

We detected both HRV-A and HRV-C among hospitalized patients. Among children, both HRV-A and HRV-C detections were epidemiologically associated with hospitalized lower respiratory tract illness among children. Among adults, HRV-A and HRV-C detections were more common among hospitalized adults with lower respiratory illness compared to controls, however, the risk measurements were not statistically significant, likely due to small numbers after stratification by species. Unlike other reports in the literature [1], [2], [16], we were unable to demonstrate that HRV-C was associated with more severe illness compared to other HRV species. HRV-A was the most commonly detected species among hospitalized adults and infants <1 year of age but HRV-C was the most common detected species among children aged 1–18 years. Our results differ from a recently publicized study of hospitalized children aged <5 years in Thailand that reported HRV-C as more common than HRV-A among children in all age groups during 2007 [30]. Studies with larger cohorts might provide additional insight into these observations. Also, additional methods to differentiate HRV, in addition to species, may help further characterize them and the illness associated with them.

Among hospitalized enrolled adults aged >50 years with lower respiratory tract illness in SaKaeo province, the rate of HRV detections with and without co-detection of another pathogen was high, second only to influenza viruses [23]. Hospitalized adults were 4-fold more likely to have HRV detected compared to controls; less than five percent of control adults had HRV detections. In addition, the rates of HRV detection among adults ≥50 years of age with radiographically-confirmed pneumonia with and without co-detection of another pathogen were similar to rates for detection of influenza viruses and *S. pneumonia* [23]. Approximately, 8% of radiographically-confirmed pneumonias among adults ≥20 years of age were associated with HRV detection (without another pathogen detected). Our HRV detection results are similar to those reported from a one year cohort study of hospitalized adults with community-acquired pneumonia in New Zealand [12]. Also, several reports have found an association with HRV detection and asthma exacerbation [5], [14], [22], [31]. Thus, treatment or prevention modalities for HRV might reduce the significant morbidity associated with HRV-associated asthma and adult lower respiratory tract hospitalization.

We estimated high rates of HRV detections among children <5 years of age. However, this age group also had a high frequency of HRV detections among young control children without respiratory symptoms or fever. Thus, our rates may not reflect the actual burden of HRV-associated illness among hospitalized pediatric patients; we cannot differentiate those children who were hospitalized with HRV-associated illness versus those that may not have had symptoms due to HRV despite HRV shedding. Several reports suggest that young children shed HRVs for more than one week [19], [20], [22]. Therefore, it is possible that HRV detection among ill or non-ill children might be from a previous illness. We did not ask controls about illness more than three days previously or follow them to see if they became ill, and no hospitalized patients were asked about prior illness unrelated to the hospitalization. Also, the presence of rhinorrhea was not an exclusion criterion for controls and was not recorded; recording rhinorrhea and previous history of illness would improve the useful of this type of control. Thus, while patients with HRV detections were associated with hospitalized lower respiratory tract illness, it is not possible to determine the actual number or burden of HRV- associated illnesses among pediatric patients. Additional studies, especially those with a longitudinally followed cohort, are needed to better characterize HRV-associated illness among hospitalized children.

Almost half of the hospitalized children with HRV detection and up to a third of hospitalized adults with HRV detection had co-detection of another respiratory pathogen. These proportions are similar to other reports [4]. In addition, previously we reported co-detection of human bocavirus and coronaviruses with HRV during the second study year [32], [33] but did not include them in this analysis due to their uncertain association with hospitalized illness. Our results support the identification, and probable exclusion, of infection with other known respiratory pathogens as important for evaluating illness possibly associated with HRV.

Our results are limited by some potential biases. We did not enroll hospitalized patients who met the clinical case definition for acute lower respiratory illness but did not have a chest radiograph ordered within 48 hours of admission or who presented with other clinical syndromes, including those with exacerbations of underlying disease. Patients with acute lower respiratory illness who did not get a chest radiograph tended to be younger and possibly less seriously ill (i.e., fewer intubations and fatalities) (HC Baggett, person. comm.). Some patients with acute lower respiratory illness who did not get a chest radiograph may have had illness due to HRV; thus, our incidence estimates for hospitalized HRV-associated illness may be underestimated. Although only 45% of eligible patients were enrolled, we found few differences in clinical characteristics between those who were enrolled compared to those who were not enrolled. However, young children and those in the intensive care unit were less likely to enroll in the etiology study (HC Baggett, person. comm.). Our study results may not be representative of all young children and seriously ill. Also, we were able to test for limited bacterial co-detections among children and did not have blood cultures. In addition, we only tested for atypical bacteria during one year. Thus, we likely missed some bacterial co-detections. Finally, our outpatient sample was a convenience sample and may not be representative of the population in Sa Kaeo Province; few adults were enrolled in the outpatient setting with influenza-like illness.

We present evidence for HRVs, and HRV-A and HRV-C, as pathogens associated with hospitalized respiratory illness and as a virus detected when there was no direct connection with illness (i.e., bystander virus). Among adults and children in rural Thailand, HRVs were associated with a substantial burden of hospitalized lower respiratory tract illness. While further studies are needed to characterize the true burden of HRV-associated pediatric hospitalizations, new treatment or prevention modalities effective against HRV would likely reduce what appears to be a substantial morbidity associated with HRV-associated illness.

## Supporting Information

Table S1 Laboratory methods used to identify respiratory pathogens.
(DOCX)
