# Supplementary material for: Human Rhinovirus Infections in Rural Thailand: Epidemiological Evidence for Rhinovirus as Both Pathogen and Bystander
Source: PLoS One. 2011 Mar 29;6(3):e17780. doi: 10.1371/journal.pone.0017780 (PMC3066183; doi:10.1371/journal.pone.0017780)
Supplement: Table S1 — Laboratory methods used to identify respiratory pathogens. (DOCX) [file pone.0017780.s001.docx]

**Table S1.** Laboratory methods used to identify respiratory pathogens

| Respiratory pathogen | Assay | Specimen type | Patients tested | Ages tested | Dates tested (if limited) |
| --- | --- | --- | --- | --- | --- |
| Rhinoviruses | RT-PCR | NP swab | Hospitalized Oupatients Controls | All ages | Sep 2003-Aug 2005 |
| Adenoviruses | Culture, RT-PCR | NP swab | Hospitalized Oupatients Controls | All ages |  |
|  | ELISA (IgG) | Serum | Hospitalized | All ages | Sep 2003-Aug 2004 |
| Influenza viruses (A and B) | Culture, RT-PCR | NP swab | Hospitalized Oupatients Controls | All ages |  |
|  | Hemagglutinin inhibition (HI) test | Serum | Hospitalized | All ages | Sep 2003-Aug 2004 |
| Metapneumovirus | Culture, RT-PCR | NP swab | Hospitalized Oupatients Controls | All ages |  |
|  | ELISA (IgG) | Serum | Hospitalized | All ages | Sep 2003-Apr 2005 |
| Parainfluenza viruses, 1-3 | Culture, RT-PCR | NP swab | Hospitalized Oupatients Controls | All ages |  |
|  | ELISA (IgG) | Serum | Hospitalized | All ages | Sep 2003-Aug 2004 |
| Respiratory syncytial virus | Culture, RT-PCR | NP swab | Hospitalized Oupatients Controls | All ages |  |
|  | ELISA (IgG) | Serum | Hospitalized | All ages | Sep 2003-Apr 2005 |
| *Chlamydia pneumoniae* | Focus Diagnostics^®^ microimmunofluorescence assay (IgG and IgM) | Serum | Hospitalized | All ages | Sep 2003-Aug 2004 |
|  | PCR | NP swab | Hospitalized | All ages | Sep 2003-Aug 2005 |
| *Legionella longbeachae* | Indirect immunofluorescence assay (IgG, IgA and IgM) | Serum | Hospitalized | >18 years | Sep 2003-Aug 2005 |
| *Legionella pneumophilia* serogroup 1 | Binax^®^ Now rapid immunochromatographic assay | Urine | Hospitalized | >18 years | Sep 2003-Aug 2004 |
| *Mycoplasma pneumoniae* | Remel^®^ ELISA (IgG and IgM) | Serum | Hospitalized | All ages | Sep 2003-Aug 2004 |
|  | PCR | NP swab | Hospitalized | All ages | Sep 2003-Aug 2005 |
| *Streptococcus pneumoniae* | Binax^®^ Now rapid immunochromatographic assay | Urine | Hospitalized | >18 years |  |

NP=nasophayrngeal, RT-PCR = reverse transcriptase - polymerase chain reaction (individual assay for each pathogen), ELISA = Enzyme-linked immunosorbent assay

*This table was modified from a previously published table. (Olsen SJ, Thamthitiwat S, Chantra S, Chittaganpitcha M, et al. Incidence of Respiratory Pathogens in Persons Hospitalized with Pneumonia in Sa Kaeo and Nakhon Phanom Provinces, Thailand. *Epidemiology and Infection.* 2010;31:1-12.)
